# Smoking in men and women with type 2 diabetes: A qualitative gender-sensitive exploration of barriers to smoking cessation among people with type 2 diabetes

**DOI:** 10.1371/journal.pone.0221783

**Published:** 2019-08-28

**Authors:** Aurélien Georges, Laura Galbiati, Carole Clair

**Affiliations:** 1 Programme cantonal Diabète (PcD), Department of Ambulatory care and Community Medicine, University of Lausanne, Lausanne, Switzerland; 2 Institute of Psychology, University of Lausanne, Lausanne, Switzerland; 3 Department of Ambulatory Care and Community Medicine, University of Lausanne, Lausanne, Switzerland; Medical University of South Carolina, UNITED STATES

## Abstract

**Objectives:**

Despite growing evidence of the impact of smoking on diabetes complications, people with type 2 diabetes still smoke at high rates and little is known about the specific barriers to quit smoking in this group. The purpose of this article is to explore the perception of smoking, and motivation and barriers to quit among smokers with type 2 diabetes. This exploratory study will help designing a smoking cessation intervention tailored to the needs of people with type 2 diabetes. We hypothesize both that living with diabetes and gender may raise additional difficulties to quit smoking.

**Setting:**

The qualitative exploratory research included ten in-depth semi-structured individual interviews and five focus groups conducted in an Ambulatory Care University Hospital in Switzerland. The thematic analysis was conducted with a gender-sensitive focus.

**Participants:**

Both current and former smokers recruited from the ambulatory clinic and the community took part in the qualitative interviews. We restricted the analysis to 21 current smokers only.

**Results:**

The sample included 12 men and 9 women with type 2 diabetes, having a mean age of 59.4 years, who had diabetes for an average of 9 years with a mean HbA1c of 7.4%. We found that harmful effects of tobacco were superficially understood, and participants used several patterns of thinking to minimize the risks. The relation between tobacco and diabetes was poorly known. Readiness to change was related to personal self-image and meaningful engagement in life and social relationships. Barriers could be organized into three groups: dependence, psychological habits and social barriers. Barriers were markedly shaped by gender and living with diabetes.

**Conclusions:**

Results suggest that “one -fits-all” smoking cessation interventions do not satisfy the needs of type 2 diabetic smokers. Personalized smoking cessation interventions should be preferred and should pursue positive psychological outcomes addressing contextual factors.

## Introduction

Smoking and diabetes act synergistically on morbidity and mortality[1, 2]. People with diabetes who smoke are at increased risk of coronary heart disease [3, 4] and microvascular complications[5, 6]. Up to 65% of cardiovascular mortality is attributable to interaction between smoking and diabetes [7]. Research suggests that diabetic people who smoke have poorer diabetes control [8, 9], greater insulin needs[10], increased insulin resistance[11] and increased risk of hypoglycemia [12].

Despite increasing evidence of the metabolic risks caused by the interplay between smoking and diabetes, prevalence of smoking remains high among people with diabetes. In a country such as Switzerland, it is estimated that 20.8% of people with diabetes are current smokers [13, 14].

Previous research suggests that people with diabetes have specific behaviors and needs towards smoking [15–20]. In a recent qualitative study among type 2 diabetic smokers in Hong Kong, knowledge of the association between smoking and diabetes was poor and type 2 diabetic smokers had negative attitudes about quitting smoking [21]. Smoking peers as well as psychological addiction and post-cessation weight gain were mentioned as barriers to quit, whereas awareness of health and family support were motivators for smoking cessation. In addition, gender might play an important role both in smoking and smoking cessation [22, 23] and in diabetes self-management [24]. Women are less likely to quit smoking than men [25, 26] and have different reasons and motivations to smoke [27, 28] such as weight management and coping with stress and emotions[27, 29, 30]. The prevalence of type 2 diabetes is higher among men [31] whereas women have a better glycemic control and adherence to recommended self-care compared to men[24]. Few studies have assessed the specific needs of type 2 diabetes smokers regarding smoking cessation and none has considered gender influence.

The purpose of this qualitative study is to gain a better insight into the interconnection of diabetes, smoking and gender to be able to design a more effective smoking cessation program. This study specifically explores the way people with type 2 diabetes related to smoking cessation in a Swiss sample of smokers with type 2 diabetes and explores specific needs and beliefs towards smoking cessation.

## Materials and methods

The current research is an exploratory qualitative study. Our approach is rooted in a realist social constructionism perspective[32, 33] and is based on the theoretical framework of the phenomenological perspective in medical anthropology (e.g. Good[34]; Csordas[35]; Bibeau[36]; Dressler[37]). Our goal was to explore individual experiences of everyday life in particular smoking, in order to understand them in a larger socio-cultural context that shapes individual opportunities and narratives.

We conducted ten in-depth semi-structured individual interviews with current and former smokers with type 2 diabetes. Interviews were used to get a deep insight into the relationship of participants with tobacco in the context of their daily experience. We then conducted five focus groups including 3 to 5 participants who were current and former smokers, in order to compare and contrast the relation to smoking and smoking cessation and to discuss emerging findings as a group. We stopped the individual interviews and focus groups after 21 participants, as we considered that saturation was achieved. We had specified in the protocol that data collection would continue until saturation point had been reached and no new domains or themes were generated.

We carried out sampling purposively, in a way that ensured even distribution of smoking status (former smoker, current smoker motivated to quit, current smoker not motivated to quit), gender, diabetes duration and educational level. These characteristics were the most crucial regarding our research interest.

We conducted interviews and focus groups in the conference rooms of the Ambulatory Care University Hospital, from August 2015 to February 2016. The interview team was formed of two investigators: the principal investigator (PI) of the study, a 39-year-old white woman, trained as a general internist and a research collaborator, of 29 years, white male, trained as a sociologist. To reduce influential bias, status, function and hierarchical position were not introduced to participants and visual indication of status was purposely removed (for example lab coat and signs of grade). No participants were under the care of the PI and it was explicitly mentioned that participation in the study was disconnected with care.

We sent a complete study description to the participants prior to the interview and researchers made sure that the participation conditions were well understood by repeating the most important conditions at the beginning of the interviews. Informed consents were signed before interviews started. The Cantonal Ethics committee (Commission cantonale (VD) d’éthique de la recherche sur l’être humain (CER-VD) http://www.cer-vd.ch) approved this study (CER-VD protocol n°302/15). Written and oral consetn were obtained from participants to this study.

Interviews and focus groups were based on interview grids. For individual interviews, the first part of the interview aimed at understanding the participant’s daily life, context and perception of life with diabetes, enabling the second part of the interview to focus on the knowledge and perception about smoking and living with diabetes, motivation to smoke and quit smoking and potential impediments to smoking cessation in the context of their everyday experience. For the whole sample we addressed questions about how gender impacts smoking, quitting smoking and managing diabetes. At the end of the interview, we gathered socio-demographic information as well as information regarding diabetes and smoking history using a short quantitative questionnaire.

Interviews were integrally audio-recorded and transcribed *verbatim*. Transcriptions and analyses were done using MaxQDA software. We checked the accuracy of the transcript during the first coding phase by the research team. We anonymized the transcript material and stored it on a secured drive.

We performed a template analysis established by King[38, 39]. We based the first code templates on the individual interview guide. After primary coding of all the data, we reviewed the whole code system to check the validity of the codes with the data. When needed we revised the template, by refining categories, reviewing hierarchical organization and clarifying labels. This was done with one member (the PI), who checked the codes and discussed them with the research collaborator. Disagreement were resolved by consensus or if needed discussion with a third collaborator. Finally, codes were organized to facilitate the clinical application. We asked three questions relevant to designing a smoking cessation intervention, consistent with the *Information-Motivation-Behavioral Skills* Model by Fisher and collegues[40]:

1. Information: What is the knowledge about harms and risks related to tobacco smoking and the interaction between smoking and diabetes?
2. Motivation: What are the motivations to quit? In particular, is diabetes a motivation to quit?
3. Behavioral skills: What are the obstacles and barriers to smoking cessation? Is diabetes a barrier?

Gender analysis was an interpretative process based on the analytic framework provided by Nentwich[41], that suggests analyzing gender as structure, hierarchy and identity, while paying attention to the contextual form of the expression of gender and its level of relevance in specific contexts.

For this last review, we checked reliability of the coding system and created summaries to prepare the reporting phase. Trustworthiness was improved through multiple confrontations between codes and data. Coding systems were reviewed by the PI and disagreement were resolved by consensus and if needed discussed with a third collaborator.

In this manuscript, we present an analytic narrative of relevant results regarding research questions. We structured the report to reach the Standards for Reporting Qualitative Research (SRQR)[42].

## Results

### Sample

The initial sample included 21 current smokers and 12 former smokers. In the present analysis we included only the 21 current smokers. Demographic characteristics and information on smoking and diabetes are summarized in Table 1. Participants were 59.4 years-old (SD 8.0), they had diabetes for an average duration of 9 years (SD 10.1), with few complications and medium glycaemic control (mean HbA1c 7.4%), according to current Swiss recommendations [43] (adapted from the the European Association for the Study of Diabetes[44] and the American Diabetes Association Guidelines—2016[45]) where target HbA1c is ≤6.5 but target HbA1c at 7–7.5 can be considered as acceptable in patients with co-morbidities or lower life expectancy. The majority of participants was not motivated to quit smoking (n = 8, 38.1%), or planned to quit in more than one month’s time (n = 6, 28.6%), with 5 participants (23.8%) planning to quit in the next month and two (9.5%) currently quitting.

**Table 1:** Participants’ characteristics.

	Women (n = 9)	Men (n = 12)	Total (n = 21)
**Age (years, mean, SD)**	58.8 (6.8)	59.8 (9.1)	59.4 (8.0)
**Employment (N, %)**			
** Employed**	7 (77.8)	2 (16.7)	9 (42.9)
** Unemployed**	0 (0)	5 (41.7)	5 (23.8)
** Retired**	2 (22.2)	5 (41.7)	7 (33.3)
**Marital status (N, %)**			
** Married/living with partner**	4 (44.4)	3 (25.0)	7 (33.3)
** Single/divorced**	4 (44.4)	7 (58.3)	11 (52.4)
** Widowed**	1 (11.1)	2 (16.7)	3 (14.3)
**Education (N, %)**			
** University**	3 (33.3)	4 (33.3)	7 (33.3)
** Secondary school**	1 (11.1)	2 (16.7)	3 (14.3)
** Apprenticeship**	3 (33.3)	3 (25.0)	6 (28.6)
** Obligatory school**	2 (22.2)	3 (25.0)	5 (23.8)
**Diabetes Complications (N, %)**			
** No**	7 (77.8)	6 (50.0)	13 (61.9)
** Yes**	1 (11.1)	6 (50.0)	7 (33.3)
** Do not know**	1 (11.1)	0 (0)	1 (4.8)
**Diabetes duration (mean, SD)**	12.6 (14.1)	6.4 (5.0)	9.0 (10.1)
**Pack-years (Mean, SD)**	34.4 (13.5)	54.6 (35.2)	47.2 (30.3)
**Motivation to quit among smokers**			
** Currently quitting**	0 (0)	2 (16.7)	2 (9.5)
** Plan to quit in < 1 month**	2 (22.2)	3 (25.0)	5 (23.8)
** Plan to quit in > 1 month**	3 (33.3)	3 (25.0)	6 (28.6)
** Not motivated to quit**	4 (44.4)	4 (33.3)	8 (38.1)
**BMI, kg/m2 (Mean, SD)**	28.2 (12.0)	27.7 (2.6)	27.9 (6.8)
**HbA1c, % (Mean, SD)**	7.9 (2.0)	7.2 (1.0)	7.4 (1.5)

Results are organized according to the three research questions described above. They are summarized in Table 2.

**Table 2:** Major findings of the qualitative study stratified by gender.

	Influence of diabetes	Gender influence	Other influences
**Information / Awareness of the risks of smoking on health in general**			
** **Lacking accurate information	-	/	Level of education Having a cardiovascular or respiratory disease
** **Not feeling concerned by harmful effects of smoking	-	M++	
** **Arguing that current patterns of smoking are protective	/	M+* F++*	sense of control on tobacco intake
Considering less at risk compared to other smokers/addictions	/	M+	
**Motivation to quit**			
** **Tobacco smell, taste, and cost of smoking	/	F+	
** **Concerns about health and impact on diabetes	+ ++ (if diabetes complications)	F++ M+/-	Readiness to quit smoking Having a cardiovascular or respiratory disease Peer experience health problem due to tobacco Hospitalization
** **Smoking is irreconcilable with positive self-image	/	M++ F+	Seeking a feeling of independence
** **Smoking is incompatible with meaningful engagements	/	M++* F+++*	
** **Social and contextual motivations to reduce or quit		M++* F+*	
**Obstacles and barriers to stop smoking**			
** **Physiological benefits of tobacco	++	/	Feeling of powerlessness Managing pain and stress (incl. due to diabetes)
** **Habits, psychological barriers	/	/	
** **Social barriers	/	M+++* F+++*	

* Gender impact is found to be relevant but this impact has different forms between genders.

The impact of living with diabetes and gender are schematically reported. A plus (“+”) indicates that diabetes or gender increase the phenomenon, a minus (“-“) indicates that the factors decrease it. Slashes (“/”) mean no influence on the basis of our data.

### 1. Information

Nobody in our sample firmly denied that there are risks related to tobacco smoking. Despite this superficial agreement on tobacco products hazard, we identified four patterns of thinking aimed at minimizing the risks of smoking.

#### Poor awareness of the health consequences of smoking

When addressing tobacco-related health problems, most participants had scarce knowledge of what is harmful in smoking and what the actual consequences of tobacco on health are. Participants were able to express only a reduced number of complications.

The best-known harmful effects of tobacco the participants reported were: respiratory problems, a decrease in the participant’s ability to undertake moderate physical activities and risks of cardiovascular complications.

A few participants reported clear knowledge on the relationship between tobacco and diabetes and in these cases their physician had informed them. Almost half of the smokers recognized that living with diabetes could cause particular health problems and that smoking could worsen them and women, more often than men expressed this concern.

People who did not perceive a relationship between smoking and diabetes were unable to perceive a logical connection between them:

“I didn’t think there was sugar…, what I mean is, **there isn’t sugar in cigarettes**.”

ID11 woman

Some participants focused on body weakening as a whole (aging), to deny a potential causal relationship between smoking and diabetes. Notably, only men expressed this conception.

“No, **I mean apart from having diabetes, I’m not too dilapidated**. […] **I don’t feel bad from diabetes**, every year my kidneys are checked, or my eyes or sensation is checked, my foot sensitivity hasn’t changed, and my eyes haven’t changed, the kidneys have had a bit of tinkering, but well… In eighteen years, **I think if I had an 18-year-old car, it would be more run-down than me!** (Laughter)”

ID08 man

These participants were likely to dismiss the relationship by arguing that diabetes would develop anyway without smoking, based on examples of family members and friends.

#### Minimizing the harmful effects of tobacco

More than half the participants considered that smoking was not a problem since they were not experiencing serious lung problems. They portrayed themselves as being “fairly” fit. In particular, three participants (2 men and 1 woman) considered themselves strong enough to contain and cancel out the potential health problems caused by tobacco.

"The doctor, who operated my heart, told me, **that we all have our strong and weak points**. One of us might have to stop smoking one might not need to. In the end it depended on our good and bad points. Well … **I stopped at that, because it suited me to”**

ID22 man

#### Smoking “the right way” or harm reduction

Many participants mentioned that the manner in which they smoked significantly reduced the harmful effects of smoking.

Both men and women participants described that they did not smoke many cigarettes a day, or currently less than previously. Some participants of both genders claimed they were not dependent on tobacco, or that they were able to take a temporary break from tobacco smoking.

Another strategy to reduce perceived harm of tobacco was typically expressed by women. They described their way of smoking as “soft”, not or less harmful and not problematic; for example, never smoking in the morning, not smoking the whole cigarette or not inhaling deeply cigarette smoke.

#### “It’s not as bad as others (people or risky behaviors)”

Misconceptions on harmful effects of tobacco were reinforced through positive comparisons. This comparison could be made either to other people, or to other risky behaviors perceived as “worse”. While these kinds of comparisons concerned both genders, a higher proportion of men tended to use these comparisons. The analogy was always made to men at higher risk.

"**I’m not addicted like my son**, well, him, in the morning he’s already at it, I tell him " you’re ridiculous! "

ID05 man

### 2. Motivation

We categorized major quitting motivation elements into five groups, organized in ascending order of scope.

#### Tobacco smoke and costs

Participants expressed displeasure or even disgust towards tobacco smoke and smell, based on personal taste. These preferences were more likely to be expressed by women. Smell, smoke and effects of smoke were found to be incompatible with the participant’s conceptions of cleanness or desired personal smell.

Among men, discomfort about the smell or tobacco smoke was most frequently found related to a state of illness and/or respiratory distress and not *per se*.

Cost of smoking was also mentioned as a motivation to quit smoking but to a lesser extent. Heavy smokers or smokers who were planning to quit smoking particularly expressed this concern.

#### Concerns about health and the impact on diabetes

At first participants expressed their concerns in a vague manner, for example by stating that smoking was “not good” or “bad”. However, subjects with these kinds of narratives rarely had a high level of preparedness to change.

Both men and women were more likely to raise concerns about actual tobacco-related health problems if they directly experienced negative impact on their body in everyday life. Men and women were both sensitive to susceptibility to respiratory and cardiovascular disorders caused by tobacco.

With respect to diabetes, men and women did not perceive a need to change their smoking habits due to diabetes if they did not perceive an impact of smoking on their diabetes management.

Only three male participants explained that living with diabetes might motivate them to quit smoking and this motivation was significantly related to negative impacts of diabetes, including erectile dysfunction.

Among women, a general concern about health was raised by the experience of living with several chronic diseases:

"No, no, I think that … It’s bad, very bad … Anyway, with diabetes, we’re already at risk for our veins clogging up, if we add another risk … when there’s already a chance, so no, I get it … […] [I know] very well that, having diseases, like for everyone, our disease, or my diseases, it’s not good, I know that. "

ID14 woman

Women also expressed particular concerns about adverse health problems due to tobacco, such as preserving a feminine voice.

Finally, we noticed that male participants became markedly motivated to quit tobacco if they witnessed harmful effects on others, especially family and friends.

#### Smoking conflicts with positive self-image

In the group motivated to reduce or quit smoking, participants thought that smoking conflicted with their values, aspirations or expectations about themselves.

Three female smokers expressed that smoking did not align with their personal values, considering it “stupid”, “ridiculous”, “silly”.

Almost half the participants at some point expressed having a problem with their dependence on tobacco. Dependence was experienced as a lack of liberty and both men and women shared a desire for independence often heard in narratives of a strong motivation to quit smoking.

"I don’t have a great opinion of myself, I try to do my best. What people expect of me at work, I work really well, and I think I’m worth it, owe it to myself, to make this gesture (quit smoking), and … I can’t manage, that’s the thing. It’s hard … No it’s terribly hard, isn’t it. "

ID04 woman

Sport-related metaphors were found among men:

“Just before smoking I think I should justify it because it’s a failure. Yes, smoking a cigarette is a failure for me. I mean I do it, I poison myself and that’s the first thing I tell myself. **It’s like someone who scored, a goal, for me, it’s a cigarette** I say, look; I ‘let in’ a cigarette. "

ID01 man

Men also mentioned experiencing embarrassment or feeling shame at the idea that smoking damages their image, typically if they were sportsmen.

#### Smoking prevents meaningful familial, professional or relational engagement

Smoking was sometimes perceived as conflicting with important plans or desires for social engagement. Participants purposefully chose to reduce, hide or attempt to quit smoking to get involved or to maintain engagement in meaningful relationships. The kind of dialogue was very often related to an actual plan to quit or actual attempt to quit.

Professional activity deters smoking: Participants reported that some work activity dissuades them from smoking, even if smoking is permitted in the workplace.

This was particularly evident in physically active professions, typically among men.

Female-dominated professions, such as a health care provider, particularly seem to influence women to quit smoking to avoid inconveniencing patients.

"[While she was studying nursing:] **I thought it was awful, caregivers who bent over you, smelling of tobacco, I said to myself, OK, how about one day, I go back to school and stop smoking**."

ID43 woman

Smoking is a barrier to positive engagements with others: About one third of participants of both genders felt uncomfortable about smoking in front of non-smokers. They would delay or stop smoking that then triggered them to realize that their smoking was problematic.

Women tended to pay greater attention to avoiding smoking in front of non-smokers than men. However, certain men showed more concern if smoking increased health problems for their partner, for example an asthmatic partner.

Parenting and Grand-parenting roles: Taking on parenting and grand-parenting roles was critical for smoking attitude. This was mentioned by a large majority of participants as strong motivation to reduce or attempt to quit smoking.

R1: Well maybe if I had a grandson or granddaughter to look after at home from time to time … Well, then I think I would stop entirely "

ID03 woman

This particular motivation to reduce smoking stems also from the desire to preserve their health to benefit from the joy of being (grand-) parents as long as possible.

We could not find a clear gender difference for this motivation, although men more often reported smoking at home in front of their children compared to women.

#### Social and contextual motivations to reduce or quit smoking

In this section, we identified discourses that described direct influence or incentive from a relationship(s) to quit smoking.

Take-up challenges: A challenge to quit launched between two male friends was very effective for inducing attempts to quit:

"So for me it’s exactly the opposite, because all my life everyone thought of me as a male **fighter, a bull** [(his astrological sign)], and you better not tell me “You can’t do it! " If someone says that to me, **it provokes me, I rise to the challenge**… "

ID10 man

For women, challenges rather come from the broader context of social influences:

"I started to [referring to quitting smoking the first time] because **it was a bit of a joke at the office**, I was in a large, open space office, **and the only smoker** … "

ID15 woman

Social support and encouragements to quit smoking: Participants reported that they wanted to reduce or quit smoking especially if their partners or close relations encouraged them to do so.

An effective motivation was to quit with one’s partner, if both partners smoked.

It is notable that a few single or divorced male heavy-smokers pointed out that finding a non-smoking woman was the only convincing way they could imagine to quit smoking.

Close friendships and the broader family also played a role in encouraging stopping smoking.

Smoking is not allowed: About two thirds of the participants noticed in that a public ban on smoking or agreement on smoke-free areas at home, encouraged them to reduce or quit smoking.

### 3. Behavioral skills: Obstacles and barriers to stop smoking

We ordered the perceived barriers to quitting smoking into 3 broad categories: physiological effects of tobacco and nicotine, habits and psychological barriers and social barriers.

#### Physiological effects of tobacco and nicotine

Out of twenty-one participants nineteen, both genders, talked about the physiological effects of tobacco on their bodies.

Tobacco dependence: Two thirds of the smoker participants described themselves as dependent on tobacco and dependence was used to explain why they kept smoking.

For example, for the large majority, dependence was used to express a lack of capacity to quit smoking, a state of powerlessness, most frequently experienced by women.

"Even though I don’t smoke a lot, I’m still an addict, since I smoke every day, worse though, I’d really have trouble stopping”

ID41 woman

Heavy smokers reported tobacco withdrawal symptoms when trying to quit smoking.

"If I don’t have a cigarette, I act like a drug addict, I don’t take drugs but I’m addicted, I look in all the drawers, I feel awful, it’s … You can’t imagine how addicted I am "

ID09 woman

« Stopping smoking makes me too nervous »: Some participants experienced increased nervousness after stopping smoking. This behavior might lead to negative social consequences, which deters motivation to quit smoking. Anger behavior was found to be different for men and women.

Men reported aggressiveness if they could not smoke. In some cases, this temperament gave rise to potential expression of violence towards others, including subordinates.

Among women, expression of aggressiveness from tobacco withdrawal was less socially accepted.

Diabetes as a barrier: In our sample a significant part of the participants suggested that diabetes might interfere with attempts to quit smoking.

First, smoking might be perceived as a way to manage glycemia. Smoking was also described as a mean to manage physical suffering caused by diabetes and its complications:

"When you wake up in the morning with a very high diabetes rate, you feel like your head doesn’t belong on your body! Because when my diabetes is high, I feel it in my head, for example, I’m dizzy, and then the only way to get my head back to normal is to smoke. And then have my morning coffee, isn’t it? "

ID08 woman

Female participants in particular expressed that living with diabetes was an additional stressor that prevented them from succeeding to quit smoking.

Yes, I really think, now, I have to stop because I have diabetes as well as a progressive rheumatoid arthritis […] but I’m having trouble, its difficult, so difficult, our diseases take a lot out of us on a daily basis, make physical activities hard, which is bad for diabetics who need to walk. Everything requires effort, there’s continual doubt, checking yourself 6 times a day, in the end it’s trying. For me it gets difficult to bear. So stopping smoking, I know, I saw the doctor again today, but … Even though I know I have to stop, I just can’t do it »

ID19 woman

Weight control after smoking cessation was not described as a barrier to quit smoking, even in the context of diabetes, despite participants knowing the risk of gaining weight and experiencing it after quitting. Participants who felt ready to quit were prepared to accept weight gain and deal with potential impacts on diabetes.

#### Habits and psychological barriers

Almost all participants reported that smoking was encompassed in some form of ritual or habits. Participants talked about the difficulties of doing without smoking.

"All day-long and at night, if I wake up at night, I smoke … I smoke a cigarette with my medicine in the morning, between my injections, doing anything and everything, I smoke one. But, it’s, it’s becoming a ritual with everything … Totally useless connections, but it’s like that. "

ID 22 man

Half of participants both men and women mentioned that refraining from smoking after meals, especially after lunch and dinner, was the most difficult part of an attempt to quit or reduce smoking. These specific cigarettes were perceived as the most important and pleasant.

Social barriers: Almost all participants mentioned that quitting smoking was difficult if smoking is entangled in significant relationships. Participants reported difficulties to quit if their partners kept on smoking.

"I tried twenty years ago, and stopped for six months, and I was married, to a woman who smoked, Gauloises too! But then, after 6 months, I went home one day and inhaled the scent of Gauloises, I said to myself, why on earth did I stop, and then just like that started again "

ID08 man

Family members, close friends or colleagues also had an impact on smoking behavior.

Peer relationships were a third social context group, especially difficult to manage for people who tried to quit. Among men, the male peer group was identified as a major cause of smoking relapse among current smokers. The activities associated with smoking were typically masculine such as partying with male friends or joining the army.

Women who attempted to reduce or quit smoking experienced difficulties in avoiding smoking during festive events attended by men and women, especially when drinking alcohol:

"Sometimes, it happens without thinking, usually in a group, where, maybe, we’re drinking, well, I don’t drink much alcohol, but from time to time an aperitif, and I noticed, then, I want to smoke straight away. "

ID04 woman

## Discussion

People living with type 2 diabetes have a certain degree of awareness and information about the general harmful effects of tobacco. However, accurate information was rarely available, even among those with the highest motivation to quit. The relationship between tobacco and diabetes was poorly understood and often denied. Most patterns of thinking discarding the risk of smoking on health seemed to be influenced by gender, with patterns of perception fitting more one gender than the other. Schematically, men supposed themselves stronger than the harmful effects or used social comparison to maintain a positive image on their tobacco intake, whereas women developed “soft” patterns of smoking.

We noticed that desire to quit related to positive self-image and meaningful engagement provided the strongest impetus for the actual quit attempts reported. Among attempts, deep gender structures can be at work. The motivations found most often among women were consistent with the traditional feminine role of caring about themselves and others. Conversely, when men engaged in reflexive thinking, it encompassed themes central to masculine gender identity (independence, professional identity, sportive identity). Gender relationships were found to be relevant in shaping reason, especially for smoking challenges or reinforcing gender roles.

Most of the barriers we found for quitting smoking have been previously described in other studies, such as tobacco dependence[46, 47], strength of habit[27] and social pressure[48]. Gender-relationships had a strong impact on social barriers, both within the household and in peer relationships. As found by Greaves[22, 23, 48], cigarettes have a specific meaning within gendered relationships. Between men, invitation to smoke is a significant part of the relationship. Men tend to invite and sometimes pressure women to smoke in mixed peer relationships. In familial relationship, male smokers might feel entitled to impose cigarette smoke on their family at home as a manifestation of male privilege. Diabetes seemed to increase the level of difficulty to quit smoking because it created an additional stress on top of the existing one, which is especially high in certain women due to gender structure. Our data did not suggest that weight gain after smoking cessation was a widespread obstacle to the ambition to quit smoking, even though some participants openly acknowledged this risk.

The interpretation of our results is limited due to the very small number of in-depth interviews and focus groups. Data generalization needs further research using quantitative methods. However, our results suggest directions for improving smoking cessation interventions.

Smoking cessation interventions should take into account that information on the relationship between tobacco and diabetes could be better communicated. In this vein, our findings suggest that personal experience of participants on tobacco effect is more influential than transfer of information by health professionals. Motivation analysis suggests that the major catalyst to help participants quit smoking is to assimilate their sense of positive self-image and meaningful engagement to life, which are both highly individual and gender structured. Both aspects could benefit from a personalized intervention approach (for example narrative approach or motivational interviewing) and positive psychology from the growing body of literature (e.g. Seligman [49]), which deals in a comprehensive way with the many aspects found as relevant in this study, including positive emotions, engagement, relationships, meaning of life. Even though we focused in this study on people with type 2 diabetes, all tobacco users should benefit from personalized smoking cessation interventions. This study highlight specific needs and bareers among smokers with type 2 diabetes which should be adressed.

Gender in its three dimensions, i.e. as a structure (more stressful lifestyle, including responsibility for child education and care, male social pressure from peers), hierarchy (submission to passive smoke at home or work) and identity (role played by a sense of toughness, professional preference) might impact smoking and smoking cessation. Therefore healthcare providers have to take gender better into account when counseling for smoking cessation and integrating contextual, relational and structural factors influencing smoking might increase its efficacy.

This research shows thatthere are gender specificities in beliefs, needs and barriers to quit smoking among type 2 diabetic smokers. However but not every aspect reveals gender patterns. In addition, typically gendered perceptions tend to be shared by participants for the other sex also (for example, concern for independence). This observation accounts for changes in social roles in the current society[50]. We also found that women and men with a migrant background or a man experiencing important suffering due to health problems, presented a very close relationship to tobacco, based on stress reduction that places the focus more broadly on social determinants of health[51] as a relevant field of analysis to improve smoking cessation interventions.
